# Targeting of *Uropathogenic Escherichia coli papG* gene using CRISPR-dot nanocomplex reduced virulence of UPEC

**DOI:** 10.1038/s41598-021-97224-4

**Published:** 2021-09-07

**Authors:** Surbhi Gupta, Parveen Kumar, Bhawna Rathi, Vivek Verma, Rakesh Singh Dhanda, Pooja Devi, Manisha Yadav

**Affiliations:** 1 Dr. B. R. Ambedkar Center for Biomedical Research, University of Delhi, New Delhi, India; 2 Department of Urology, University of Alabama at Birmingham, Hugh Kaul Genetics Building, Birmingham, AL USA; 3 Stem Cell Laboratory, SMiLE Incubator, Scheelevägen 2, Lund, Sweden; 4 CSIR-Central Scientific Instruments Organisation, Sector-30C, Chandigarh, India; 5 Department of Clinical Sciences, Lund University, Malmö, Sweden

**Keywords:** Biotechnology, Microbiology, Molecular biology

## Abstract

Urinary tract infections (UTI) are the most common infectious diseases in the world. It is becoming increasingly tough to treat because of emergence of antibiotic resistance. So, there is an exigency to develop novel anti-virulence therapeutics to combat multi-drug resistance pathogenic strains. Clustered Regularly Interspaced Short Palindromic Repeats (CRISPR) discovery has revolutionized the gene editing technology for targeted approach. The greatest obstacle for CRISPR/Cas9 is cargo delivery systems and both viral and plasmid methods have disadvantages. Here, we report a highly efficient novel CRISPR based gene editing strategy, CRISPR-dots for targeting virulence factor Fimbrial Adhesion (*papG* gene), the bacterial adhesion molecule. Carbon quantum dots (CQD) were used as a delivery vehicle for Cas9 and gRNA into CFT073, a UPEC strain. CQDs were covalently conjugated to cas9 and *papG*-targeted guide RNA (gRNA) forming a nanocomplex CRISPR-dots (Cri-dots) as confirmed by DLS and transmission electron microscopy. Cri-dots-*papG* significantly targeted *papG* as demonstrated by decrease in the expression of *papG*.Further *papG* deficient UPEC had significantly reduced adherence ability and biofilm forming ability as demonstrated by fluorescence microscopy and scanning electron microscopy. Also, *papG* deficient UPEC had reduced virulence as shown by significantly increased survival of *Caenorhabditis elegans* (*C. elegans*) worms compared to UPEC. Our findings suggest that targeting of *papG* gene using Cri-dots nanocomplexes significantly reduced the pathogenicity of UPEC. Thus, Cri-dots nanocomplex offer a novel anti-bacterial strategy against multi-drug resistant UPEC.

## Introduction

Urinary tract infections (UTIs) are one of the most common bacterial infections acquired in community and healthcare settings^1,2^. Although UTIs affects both men and women, it is more severe in women. 50% women are estimated to be infected at least once in their lifespan. Uropathogenic *Escherichia coli* (UPEC) are one of the most prevalent uropathogen responsible for > 80% of the cases of UTIs in humans. UPEC cause the majority of asymptomatic bacteriuria, infection in bladder (cystitis), infection in kidney (pyelonephritis) and catheter-associated UTIs^3–5^. UPEC possess repertoire of virulence and fitness factors that enable them to invade, adhere and colonize the urinary tract.

Virulence factors involved in disease progression include adhesins (P fimbriae, and type 1 fimbriae), toxins and iron-acquisition systems^6–8^. The adhesins expressed by UPEC plays a pivotal role in early stages of infection, initiating adherence to host cells, colonization and bacterial-bacterial interactions^9,10^. P fimbriae, a heteropolymeric organelle expressed by UPEC, mediates the binding to di-galactoside moiety present in the urinary tract epithelium^11–13^. P-fimbriae are encoded by the *pap* (pyelonephritis-associated pili) operon, including *papG*, which encodes the tip adhesin^13^. Epidemiologic studies in adults and children over many years have demonstrated that *papG* are predominantly present in strains causing acute pyelonephritis^14,15^. Three distinct classes (I–III) of Pap-G adhesins has been reported so far, but *papG*-II enhance the early establishment and colonization of *E. coli* infection in human kidney^16,17^. Studies have shown that *papG* is important for *E.coli,* causing recurrent UTIs in women^18^. The global emergence of antimicrobial resistance (AMR) in uropathogens during the last decade has complicated the UTI treatment^19,20^. Hence, there is an urgency to develop an alternate treatment strategy. Anti-virulence therapeutic strategies can selectively target uropathogens sparing the commensal bacteria^21,22^.

CRISPR-Cas9 system has emerged as one of the foremost technology for programmable genome-engineering tool for targeting and manipulation of genomic sequences in pathogens and mammals^23–25^. CRISPR-Cas9 system was programmed to resensitize the bacteria by targeting AMR-encoding plasmids genes involved in biofilm formation and virulence^26–28^. However, the major hurdle for the application of CRISPR–Cas systems as antimicrobial agents is the lack of an effective and specific delivery method^29^. Although viral methods are the most common CRISPR/Cas9 delivery vehicle, but they have several drawbacks such as potential off‐target effects and risk of immunogenicity^30^. Recently Nanocomplexes have been successfully used for delivery of CRISPR–Cas in both bacteria^31^ and humans^32^.

In this study, we have exploited carbon quantum dots (CQDs) as a potential delivery vehicle for CRISPR—Cas gene editing system in UPEC. CQDs are nanoparticles with a size less than 10 nm and have special optical properties due to quantum confinement^33^. Because of its excellent biocompatibility, CQDs have emerged as a potential candidate for theranostic application^34^. Also, carbon dots are proved to be potential alternative for fluorescence-based cell-labelling assays as they are non-toxic and cell internalization occurs quickly^35^. We applied the Cri-dots editing strategy to target virulence factor, *papG* in UPEC, important for adhesion to host. The establishment and application of Cri-dots editing strategy would improve the genetic engineering in bacteria and provide insights for the development of anti-virulence strategy in other bacterial pathogens.

## Results

### Carbon quantum dots (CQDs) were not cytotoxic to human cell lines

**Figure 1:**
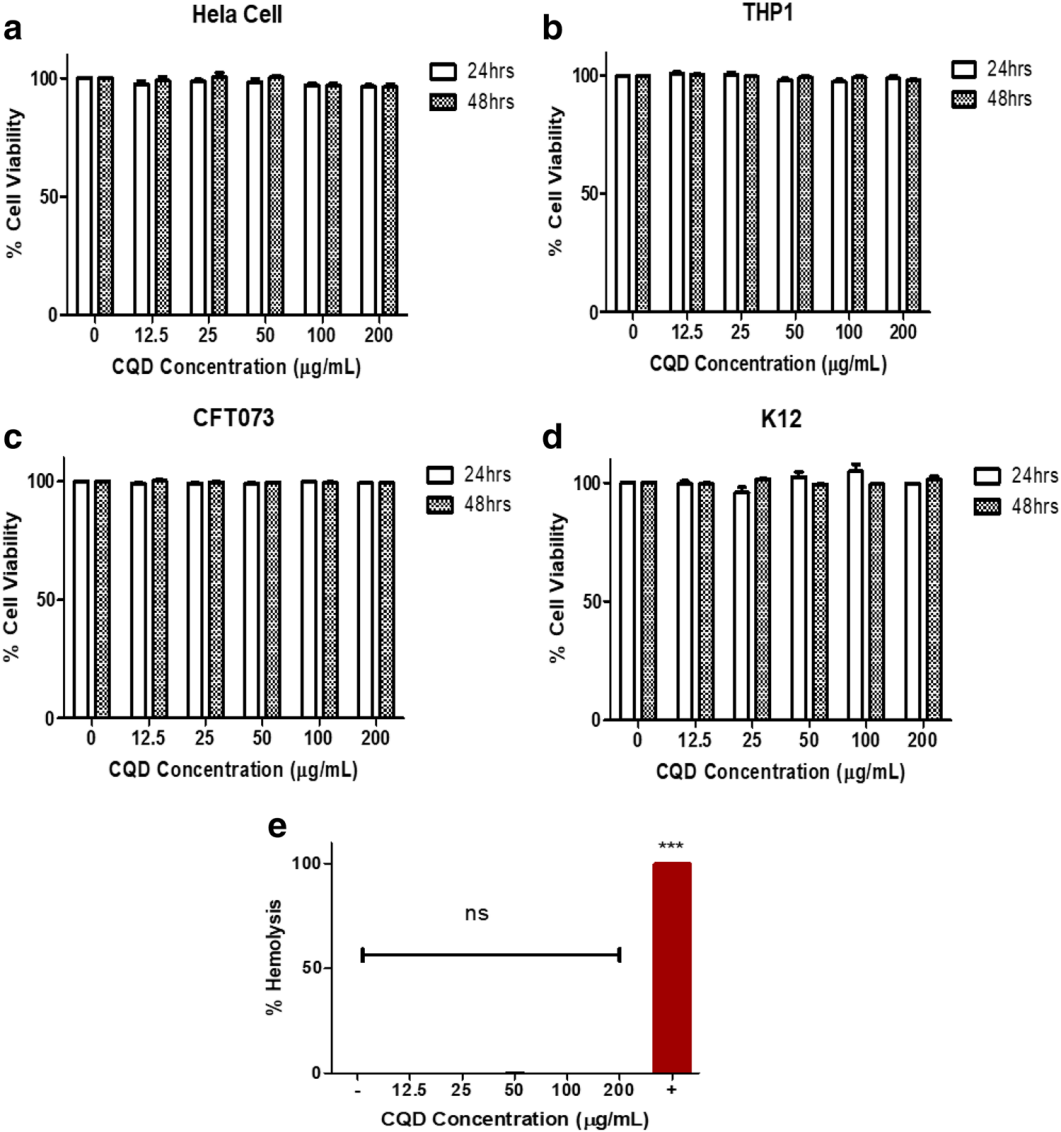
CQDs display no cytotoxicity to both bacterial and human cells. Effect of carbon quantum dots (CQDs) on the viability of (**a**) Hela cells, (**b**) THP-1 cells (**c**) UPEC strain, CFT073 and (**d**) commensal strain K12. Cells were incubated with CQDs at different doses (i.e. 0, 12.5, 25, 50, 100 and 200 ug/ml) and proliferation was observed after 24 h and 48 h using MTT assay. The data represents an average of triplicate experiments as mean ± SEM. (**e**) CQD does not induce haemolysis in RBCs. Haemolysis assay of RBCs was performed with different concentration of CQDs, using PBS as negative control ( −) and 1% triton X as positive control ( +). The data represents an average of triplicate experiments as mean ± SD (one-way ANOVA, ns-not significant, *p* ~ 0.36).

Quantum dots have gained a lot of interest in nano-theranostics applications such as sensors, drug delivery and biomedical imaging. For therapeutics purpose, CQDs should be biocompatible and show low toxicity^36^. MTT proliferation assays were performed to evaluate the cytotoxicity of the CQDs on HeLa and THP-1 cells. The viability of both HeLa and THP-1 cells remained above 95% at CQD concentrations, ranging from 12.5 to 200 μg/ml post 24 h- and 48 h- of treatment (Fig. 1a,b). The results showed that CQDs have no considerable cytotoxicity in the human cells lines used.

### CQDs display no antibacterial properties and no haemolytic ability

CQDs toxicity was evaluated on *E. coli*. i.e., CFT073 (uropathogenic strain) and K12 (non-pathogenic strain) by performing MTT assay. CQDs did not show any observable effect on the viability of bacteria. The cell viability of CFT073 and K12 bacteria remained above 98% after 24 h and 48 h of treatment, with CQD concentrations ranging from 12.5 to 200 μg/ml (Fig. 1c,d). Agar well diffusion method is used to evaluate the antimicrobial activity. Supplementary Fig. S1a and S1b shows the photographic images of agar plates with bored wells inoculated with CFT073 and K12. Overall, the data indicated no inhibitory activity of CQDs against CFT073 and K12 strains.

No obvious haemolysis of RBCs by CQDs were detected at various concentration CQDs (12.5, 25, 50, 100 and 200 μg/ml) (Fig. 1e and Supplementary Fig. S1c). The haemolysis percentage measured at the highest concentration of CQD (200 μg/ml) was less than 1% (0.0446 ± 0.008). Also, CQDs did not inhibit growth and biofilm forming ability of both CFT073 and K12 (Supplementary Fig. S2 and Supplemental Note S1). Lack of haemolytic activity and cytotoxic activity in human cells showed biocompatibility of CQDs.

### Characterization of CQD and CQD-cas9 using TEM and DLS

**Figure 2:**
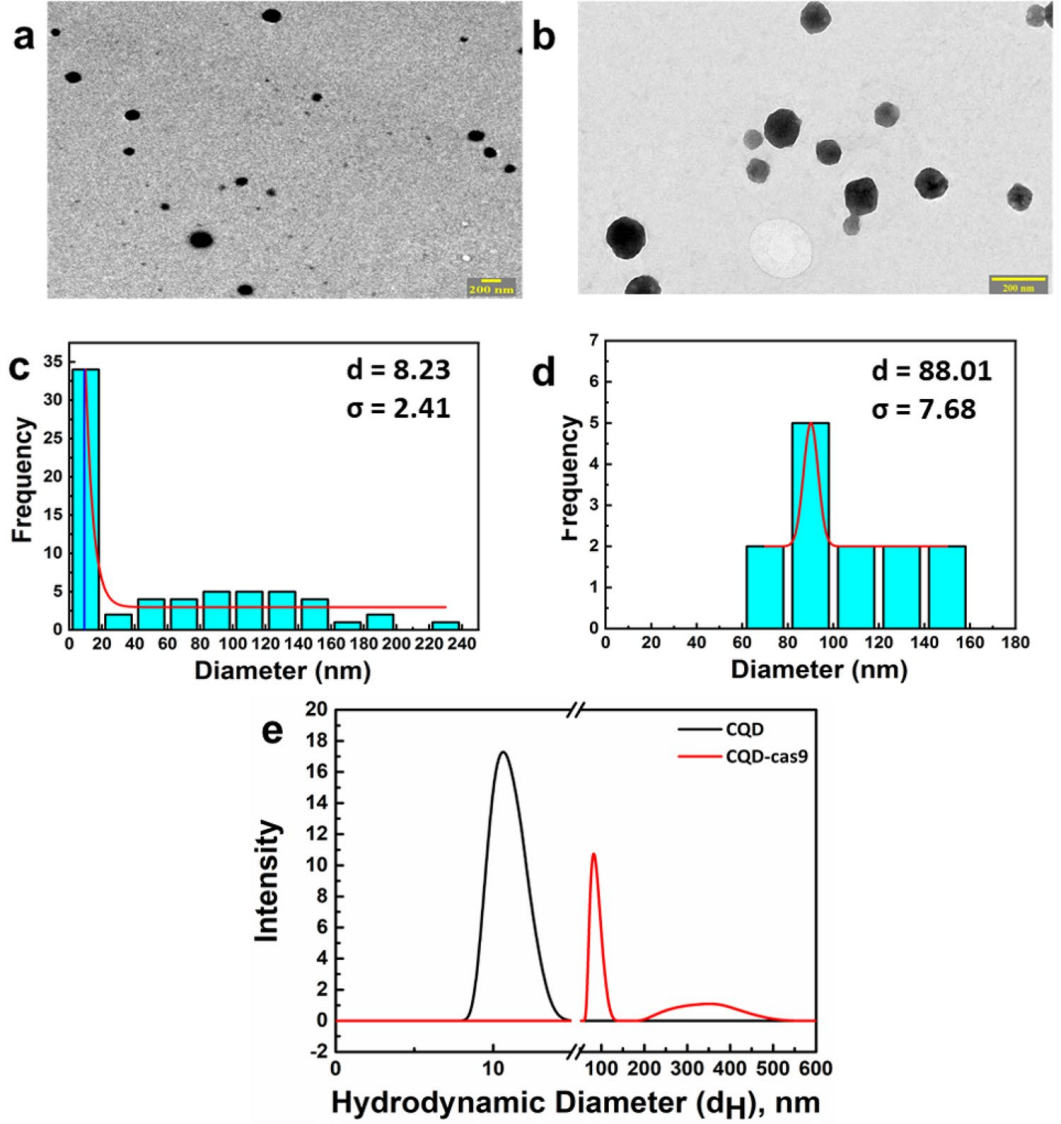
TEM micrographs and DLS for size determination. TEM micrographs of (**A**) CQDs and (**B**) CQD-cas9. (**C**) and (**D**) represents frequency size distribution bar graph for corresponding TEM images. (**E**) Size distribution of CQD and CQD-cas9 particle using Dynamic Light Scattering (DLS), d = mean diameter and σ = standard deviation.

The synthesis of CQD and CQDs-cas9 was analysed using transmission electron microscopy (TEM) and dynamic light scattering (DLS). Size measurements were performed before and after conjugation to check the increase in the hydrodynamic diameter of CQD-cas9 complex. Average size distribution data of the TEM demonstrated that size of CQDs was 8.23 ± 2.41 nm and size of CQDs-cas9 was 88.01 ± 7.68 nm (Fig. 2a,b). Figure 2c,d represents frequency size distribution bar graph for corresponding TEM images. DLS showed an increase in size of CQDS (10.86 ± 1.05 nm) to CQD-cas9 (89.31 ± 15.16 nm) (Fig. 2e). DLS plot of CQD-cas9 sample showed another small peak at 330 ± 30.64 nm, which implies the presence of some aggregates within the sample. The polydispersity index (PDI) of CQD and CQDs-cas9 conjugate is calculated to be 0.3 and 0.6, respectively. The values of PDI suggest the higher monodisperse nature of CQDs and increase in PDI of Cri-dots qualifies the complex formation with varying disperse nature.

### Cri-dots-*papG* efficiently inhibited the expression of *papG* in CFT073

**Figure 3:**
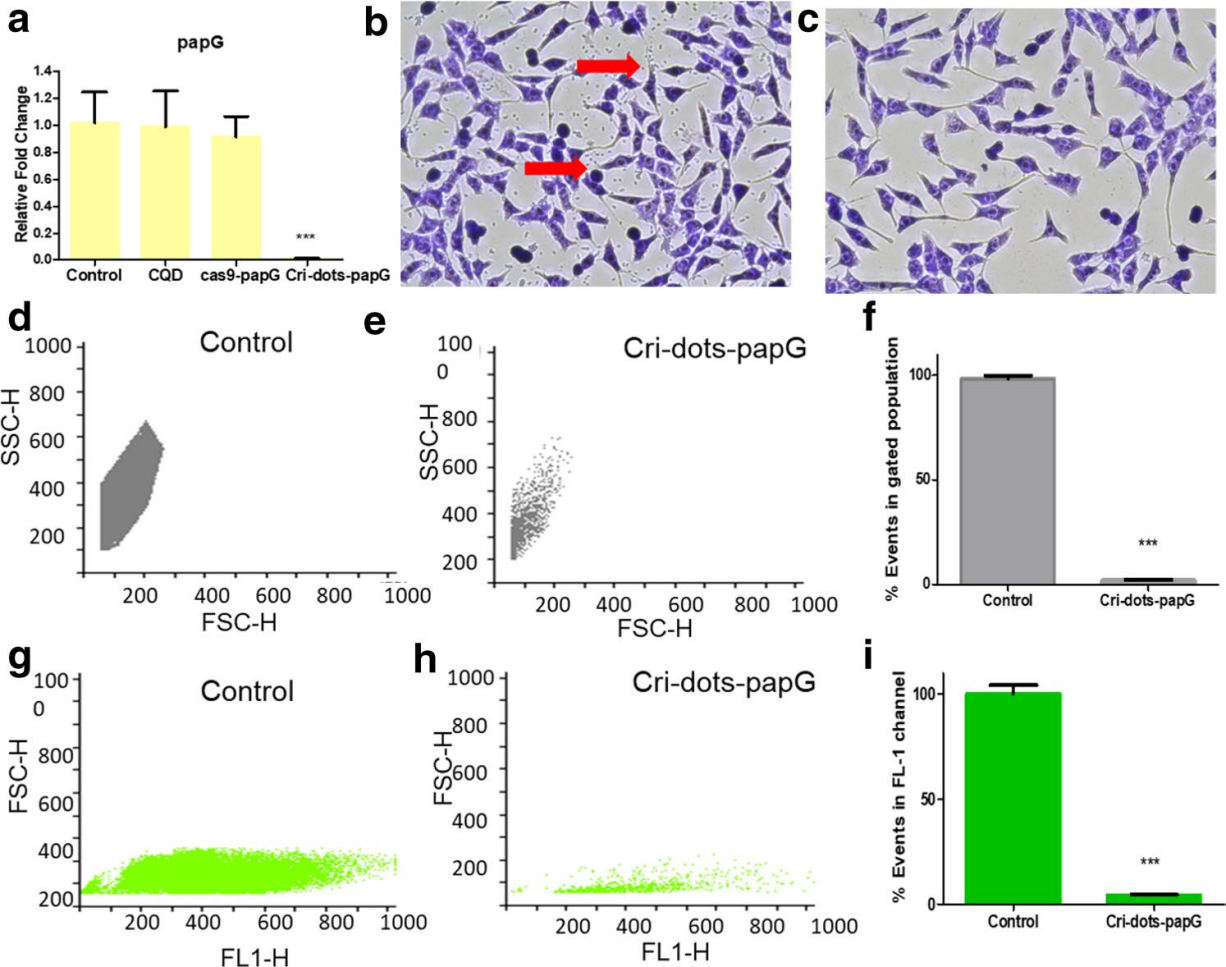
Cri-dots-papG targeting CFT073 inhibited expression of papG and reduced its adherence property. (**a**)The relative fold change of *papG* mRNA in control CFT073 and Cri-dots-papG (1 ± 0.2277, control vs 0.01049 ± 0.002956, targeted). CFT073 treated with only CQDS and cas9-papG showed no significant difference in *papG* m RNA expression.The data represents an average of triplicate experiments as mean ± SD. (***p < 0.0001, *t* test, 2 sided). Adherence Assay (**b**, **c**). HeLa cells were infected with (**b**) Control CFT073 and (**c**) Cri-dots-papG CFT073 at MOI of 1:5. At 3 h post-infection, the association of bacteria with HeLa cells was visualized by Giemsa staining. Arrows indicate bacteria associated with the HeLa cells. The plates were examined at 20 × . Flow cytometric analysis of adhesion property of Control and Cri-dots-papG CFT073 to Hela cells (**d**–**i**). Total numbers of events were acquired in 12 µL of sample volume in 60 s for each FCM analysis. Panels represents dot plots of gated population of SYTO9 stained cells (**d**, **e**) and unstained cells (**g**, **h**). Panel (**f**) and (**i**) represents the corresponding % of number of events of bacterial cells normalized to control. The data represents an average of triplicate experiments as mean ± SD. (***p < 0.001, *t* test, 2 sided).

P-fimbriae (*papG*) gene was targeted in CFT073 using CQD-Cas9-gRNA (Cri-dots-*papG*) complex. The mRNA expression of *papG* gene was evaluated by qRT-PCR to check the efficacy of Cri-dots mediated gene editing in CFT073 (Fig. 3a). Data showed that mRNA level of *papG* was drastically reduced in Cri-dots-*papG* targeted CFT073 strain compared to control CFT073 (1.0 ± 0.2277, control vs 0.01049 ± 0.002956, targeted; *p* < 0.01).

### *papG* targeting reduced adherence of UPEC

P-fimbriae are fundamental for bacterial adherence to the uroepithelium through the Galα1-4Gal-restricting *papG* adhesin^37^. Therefore, a qualitative analysis of adherence potential of control and *papG* targeted CFT073 strain to HeLa cells was investigated with the help of light microscopy (Fig. 3). The rod-shaped CFT073 were observed adhered to HeLa cells in the typical diffuse manner, (see arrow, Fig. 3b). Whereas, significantly fewer bacteria were visibly adhered to HeLa cells, infected with Cri-dots-*papG* (Fig. 3c). This showed a perceptible reduction in adherent ability of bacteria after CRISPR targeting of *papG* gene. Also, we observed *Cri-dots-papG* CFT073 had no significant effect on the morphology of HeLa cells as compared to control CFT073 (Supplementary Fig. S3).

In addition, we quantified the adherence potential of control and Cri-dots-*papG* CFT073 strain to HeLa cells by flow cytometry. Total number of events acquired in 12µL of sample, for both unstained and Syto9 stained cells obtained after adherence to HeLa cells, were calculated. There was a significant decrease in the number of the Cri-dots-*papG* CFT073 as compared to control CFT073 (*p* < 0.001). The percentage of unstained Cri-dots-*papG* strain was 2.157 ± 0.2069 as compared to adhered control strain obtained, which was 98.20 ± 1.556 (Fig. 3d–f). Similar results were obtained when bacterial cells were stained with SYTO9 (Fig. 3g–i). A drastic reduction in adherence property of Cri-dots-*papG* CFT073 strain (100.1 ± 4.115% Cri-dots-*papG* vs 4.270 ± 0.2096% control) was observed. Supplementary Fig. S4 represents the flow cytometric gating strategy of the unstained CFT073.The results were consistent in T24 urinary bladder epithelial cell lines both checked by giemsa staining and flow cytometry showing more than 90% reduction in adherence (Supplementary Fig. S5).

### *papG* targeted CFT073 showed mannose sensitive haemagglutination (MSHA)

The expression of the PapG II adhesin was compared in the CFT073, Cri-dots-papG, and K12.As established PapG II adhesin exhibit mannose-resistant hemagglutination (MRHA) while fimH adhesion show mannose-sensitive hemagglutination (MSHA) to human blood type OP1^17^. As expected CFT073 agglutinated human blood type OP1 both in presence and absence of mannose indicating MRHA whereas Cri-dots-papG did not agglutinate erythrocytes in presence of mannose which showed MSHA. These results were consistent with the K12 strains. The results confirmed the absence of papG in Cri-dots-papG targeted CFT073. In order to estimate the effect of mannose on adherence, we tested bacterial adherence to T24 cell line with untreated(-) and mannose pre-treated( +) Control and Cri-dots-papG targated strains (Fig. S6). In concurrence with our observations from the haemagglutination assay, Control CFT073 demonstrated adherance (64.61 ± 1.002 N%) in presence of 1% mannose aa compared in absence of mannose, but there was only a slight change in adherence of Cri-dots-papG (0.9610 ± 0.1068%) in presence and absence of mannose as adherence is already reduced to significant levels after papG targeting.

### Cri-dots mediated editing of *papG* reduced the biofilm forming ability of CFT073

Expression of *papG* has been reported to be prevalent in strong biofilm producers^38,39^. We estimated whether Cri-dots mediated *papG* gene editing was affecting the biofilm forming ability of CFT073. A quantitative analysis of biofilm formation ability of the Cri-dots-*papG* CFT073 was performed by crystal violet assay (CV) (Fig. 5a). The observed CV value of Cri-dots-*papG* CFT073 was significantly lower as compared to control CFT073 (0.570 ± 0.019, Cri-dots-*papG* vs 1.072 ± 0.043, control) (*p* < 0.0001). These data showed that Cri-dots mediated editing of *papG* gene in CFT073 significantly reduced their biofilm forming ability. Moreover, there was no effect seen on biofilm forming ability of K12 treated with Cri-dots-*papG* as K12 does not have *papG* gene (Supplementary Fig. S6). This further proves the specificity of cri-dots-*papG*.

Quantitative measurements of CV assay were further supported by florescence microscopy **(**Fig. 5c,d). The difference in intensity, because of SYTO9 staining of bacterial cells, showed the noticeable reduction in the biofilm forming tendency of CFT073 after Cri-dots mediated *papG* gene targeting. Similar results were observed from a quantitative analysis of the florescence images (Fig. 5b), where quenching was observed in Cri-dots-*papG* CFT073 as compared to control CFT073 (0.310 ± 0.1504, Cri-dots-*papG* vs 24.125 ± 1.2996, control) (*p* < 0.0001). These data confirm that the Cri-dots mediated editing of *papG* reduced the biofilm forming ability of CFT073.

### SEM confirmed a reduced biofilm forming capability in *papG*-targeted CFT073

Bacterial adhesins (Flagella and pili) are known to be directly involved in the attachment to abiotic surfaces in Gram-negative bacteria^40^. The effects of *papG* targeting on CFT073 biofilms forming ability and adhesion capacity were further investigated using SEM. The bacteria in the control CFT073 were observed to be encased within a self-produced exopolymeric matrix forming strong biofilm as comparison to the *Cri-dots-papG CFT073*, which showed a significant reduction in their number (Fig. 5e,f). In addition, control CFT073 had a higher number of adhered cells, while *Cri-dots-papG CFT073* had individual bacterial cells.

### Decreased Virulence of CFT073 after targeting *papG* using Cri-dots

We further evaluated the virulence of CFT073 and Cri-dots-*papG* with an in vivo *C. elegans* infection model. This was done to assess the cumulative importance of our results on the virulence of CFT073 after using Cri-dots targeting *papG*. We found that Cri-dots-*papG* significantly increased the survival of *C. elegans* worms compared to CFT073 (Fig. 6 and Fig. S7).

## Discussion

UTIs are widespread around the globe and around 150 million people develop UTI every year, resulting in high social costs^41,42^. It is estimated that 11% of women report at least once UTI in their lifetime, and the prevalence in women over 65 years of age is approximately 20%^43,44^. The introduction of the antibiotic therapy had contributed significantly to the UTIs management, but antibiotic resistance in the UPEC causing UTIs has further worsened the treatment options. Because of the global emergence of multi-drug resistant uropathogens, it is imperative to embark on parallel strategies that target specific virulence systems of bacteria to combat pathogenesis. However, some potential drug targets of uropathogens such as adhesins, toxins, capsule, urease, iron metabolism and motility have been explored, but clinical studies are still in progress^22^.

Bacterial adhesion to urothelial cells is a key step in pathogenesis of UTIs. One large family of adhesive organelles are pili assembled by the chaperone-usher pathway (CUP) pili. The most studied of the CUP pili of UPEC are the Type 1, P, and S pili that mediate microbial attachment to host tissues and biofilm formation^45^. P fimbriae are the second most common virulence factor of UPEC, which plays an important role in the pathogenesis of ascending UTIs and pyelonephritis in humans (Bien et al., 2012). P-fimbriae are encoded by the *pap* (pyelonephritis-associated pili) operon and encoded by protein subunits such as PapA, PapD, PapE, PapF, and PapG^10^. Previous studies had investigated the efficacy of vaccine containing PapA subunit, but failed due to poor generation of adherence-inhibition antibodies *in-vivo*^46,47^. Purified PapDG vaccine protected cynomolgus monkeys from pyelonephritis, however no further studies were conducted^23,48,49^. Here, we designed a novel non-viral CRISPR-cas9 gene editing strategy by Cri-dots for targeting virulence factor, *papG* in UPEC.

Recent work on the CRISPR adaptive bacterial immune system has led to the identification of new RNA-guided DNA-binding platform that can be reprogrammed to target transcription of many genes. It only requires a single protein, and a customized sgRNA designed with a complementary region to the gene of interest^50^. Many studies have used Cas9-directed cleavage at the targeted genomic site for manipulating bacterial systems^25,51^.

Several approaches have been used, including plasmid, viral, bacteriophage and nanoparticle for delivery of CRISPR-cas9. Genetically encoded phage genomes were used to deliver CRISPR-cas9 antibacterial into bacteria, but challenges arise because of the varying size and structure of different phages^52^. Although viral delivery methods are very promising, potential off‐target effects and risk of immunogenicity arise due to long-term exposure in vivo^53^. Plasmid methods have been reported for bacterial gene editing^54^, but limitations lie in off-target effects and delivery time^55^. The advantage of nonviral delivery of the Cas9 protein and sgRNA into mammalian and bacterial cells is the widely studied strategy in recent years because of its specificity, minor stimulation of immune response, and minimal exposure to nucleases^32,56^. Also, covalent conjugation of cas9 and delivery vehicle gives an advantage over non-covalent conjugation^31^.

In order to address these challenges, we developed a non-viral delivery method, Cri-dots. We used carbon quantum dots (CQDs) as a potential delivery vehicle for gene editing because of their biocompatibility and small sizes. CQDs were found to be non-toxic to both bacterial and mammalian cells. CQDs were further covalently conjugated with cas9 using EDC/NHS chemistry. Further sgRNA was also complexed with cas9-CQD conjugate to form Cri-dots nanocomplexes (Fig. 7a). Cri-dots are being reported for the first time as a targeted anti-bacterial therapy. Cri-dots could be an easy and efficient gene targeting strategy as compared to conventional plasmid and bacteriophage-based methods.

**Figure 4:**
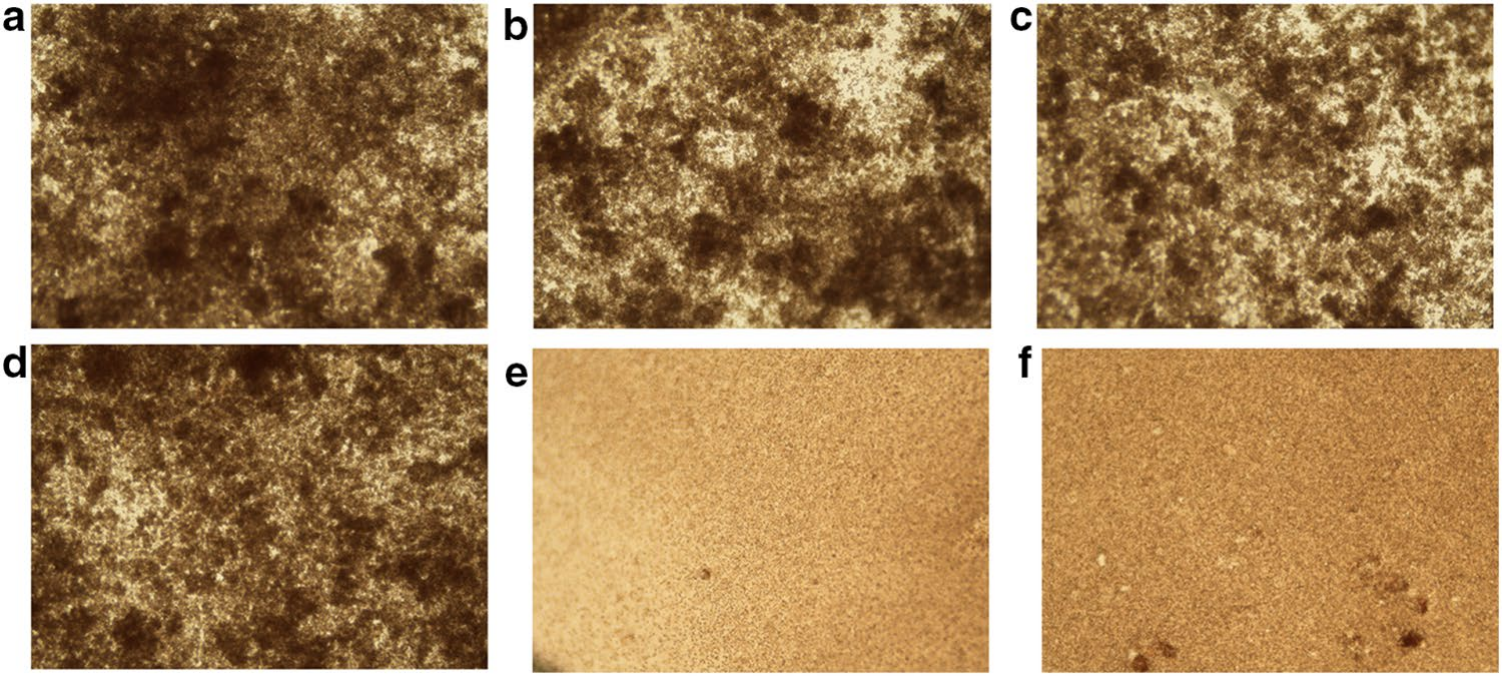
Haemagglutination analysis. Erythrocyte’s agglutination tests of the CFT073, Cri-dots-papG, and K12 in presence ( +) and absence of mannose ( −). Upper panel shows without mannose treatment (**a**–**c**) and lower panel with mannose treatment (**d**–**f**). (**a**, **d**) CFT073, (**b**, **e**) Cri-dots-papG and (**c**, **f**) K12.

**Figure 5:**
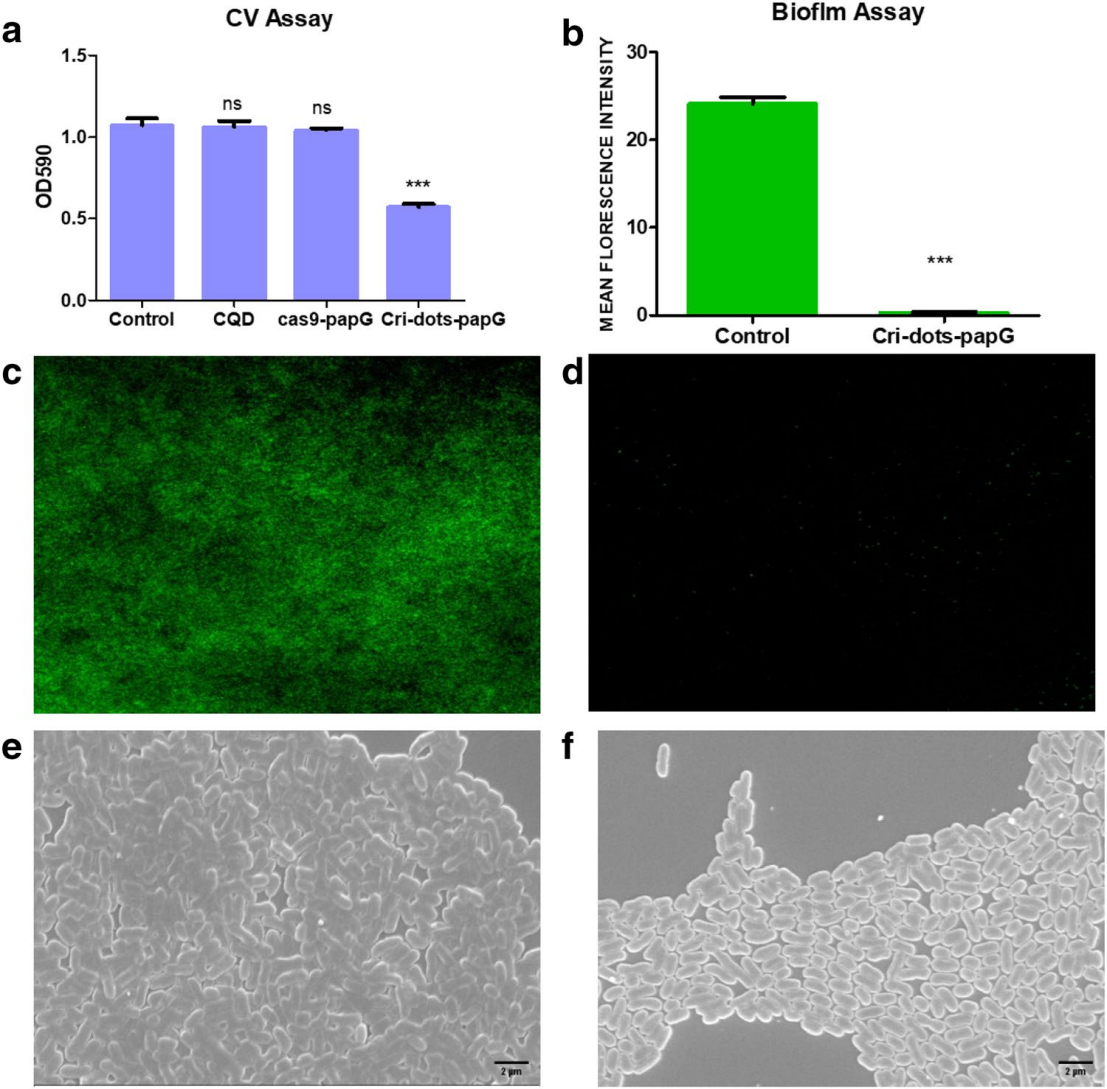
Cri-dots-papG targeting CFT073 reduces its biofilm forming ability. (**a**) Quantification of biofilm formation was assessed in control, only CQD, cas9-papG and Cri-dots-papG by crystal violet assay. The data represents an average of triplicate experiments as mean ± SD (1.072 ± 0.043, control vs 0.570 ± 0.019, Cri-dots-papG). (***p < 0.001, *t* test, 2 sided). Biofilm formation of only CQDS and cas9-papG showed no significant difference from control. Biofilm assay formation measured using florescence microscopy in (**c**) control and (**d**) Cri-dots-papG.Green fluorescence indicates Syto9 stained live bacteria. (**b**) Quantitative analysis of florescence images using imageJ and Graphpad prism5 (24.125 ± 1.2996, control vs 0.310 ± 0.1504, Cri-dots-papG). Scanning electron micrographs of (**e**) Control and (f) Cri-dots-papG.

**Figure 6:**
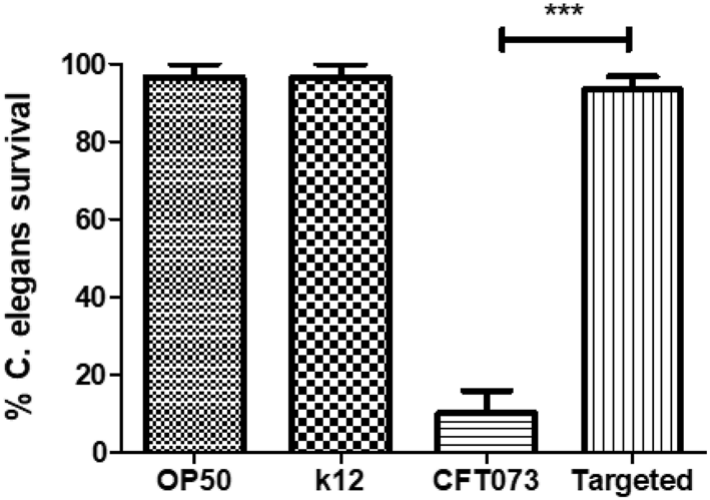
CFT073 mediated *C. elegans* killing assay. Percentage survival of *C. elegans* is represented against OP50, K12, CFT073 and Cri-dots-papG CFT073 isolates. Data are presented as mean ± SEM of 3 independent experiment.

Previous studies have shown that *papG* mutant failed to colonize or cause inflammation in kidney and are crucial for microbial adherence^57,58^. P fimbriae is strongly associated with pathogenic strains, with at least 70–90% of acute pyelonephritis in contrast to another type 1 fimbriae, which can be expressed by both commensal and uropathogenic *E. coli*^14,59^. Thus, we have chosen *papG* for CRISPR-cas9 targeting using this novel strategy involving Cri-dots for direct delivery of cas9 protein and sgRNA into the bacteria. Before proceeding with the targeting strategy, we checked the cytotoxicity of of CQDs for mammalian and bacterial cells. CQDs showed excellent biocompatibility with both mammalian and bacterial cells (Fig. 1). Further, CQDs were successfully conjugated with cas9 protein using EDC/NHS chemistry (Fig. 2). The targeting efficiency of Cri-dots was evaluated by studying the gene expression levels of papG gene in control and Cri-dots targeted cells. We observed drastic reduction in mRNA expression of *papG* in Cri-dots-*papG* targeted CFT073 strain, compared to control CFT073 (Fig. 3a). PapG has been found to be indispensable for the adherence of UPEC to epithelial cells for establishing early growth in urinary tract^16^. Hence we evaluated the adherence of papG targeted CFT073 to human cells. As expected, we have observed 96% reduction in adherence of *papG* targeted CFT073 as compared to control (Fig. 3). Moreover, papG targeted CFT073 showed mannose sensitive hemagglutination (MSHA) while control CFT073 showed Mannose resistance hemagglutination(MRHA) (Fig. 4). P fimbriae are involved in the adhesion of bacteria to mucosal epithelial through TLR4 and hence activation of immune response causing inflammation and pain^60^. Our results showed that Cri-dots targeting *papG* gene quashed the adherence ability of bacteria to a supreme extent. Thus Cri-dots-papG might reduce the virulence of UPEC to a greater extent. Interestingly, Cri-dot-papG targeted bacteria showed significant reduction in biofilm formation. Bacteria were seen scattered in papG targeted CFT073 as compared to control where dense layer in typical diffused pattern was observed (Fig. 5). Furthermore, in order to comprehend how the respective virulence alteration induced by *papG* targeting contributes to the virulence of CFT073, an in vivo* C. elegans* infection model was used. We found that Cri-dots *papG* targeted bacteria did not significantly alter the survival of *C. elegans* worms as compared to control CFT073 (Fig. 6). These results not only validate the Cri-dots as novel vehicle for targeting CRISPR-cas gene editing strategy but also propose papG as a good candidate for anti-virulence therapy against UPEC involving ascending UTIs and pyelonephritis.

**Figure 7:**
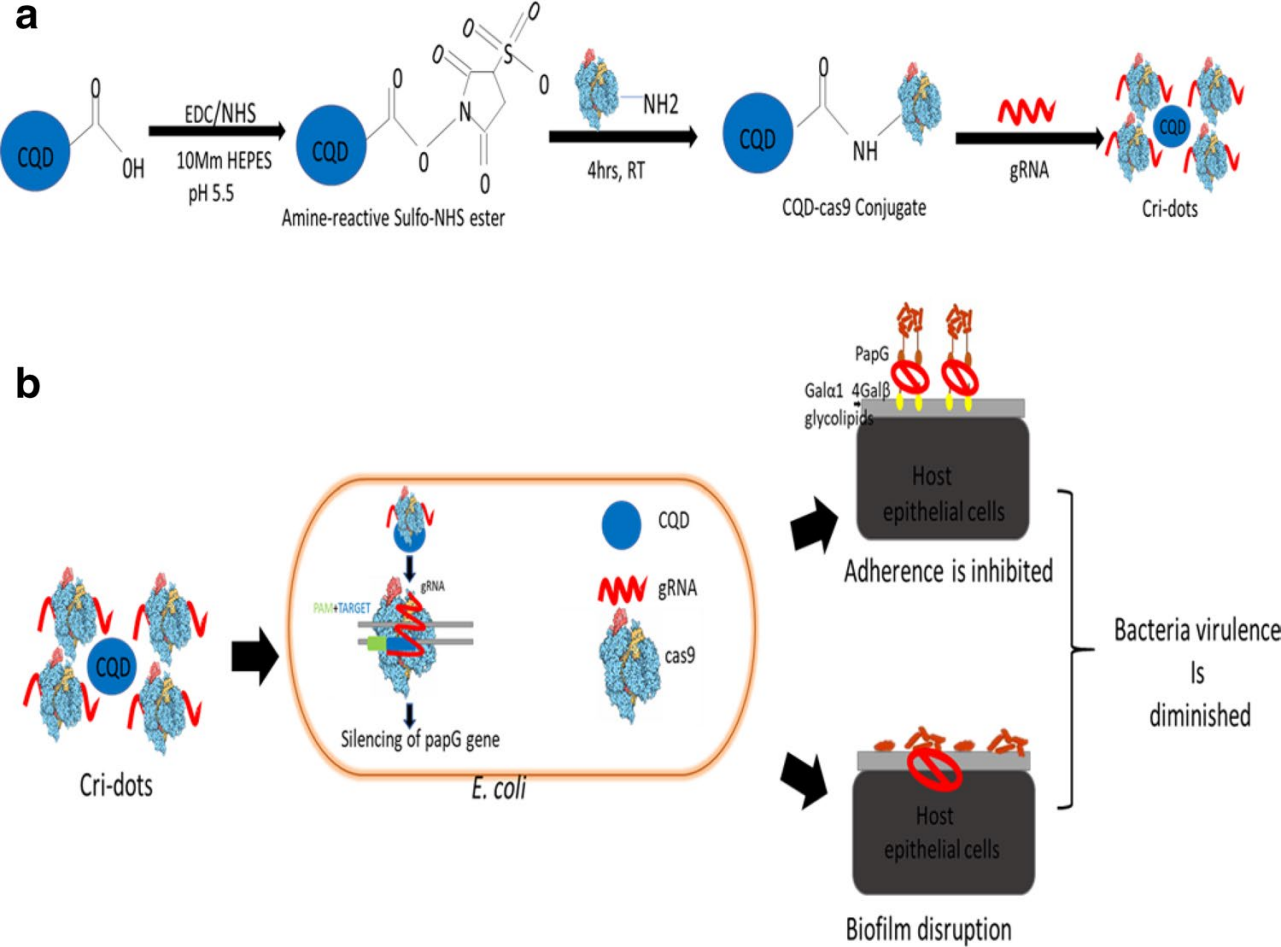
Overview of Cri-dots editing of *papG* in CFT073. (**A**) Schematic representation of amide bond formation between CQD and cas9 using EDC/Sulfo-NHS as a cross-linker to form a stable CQD-cas9 conjugate. (**B**) The representation of *papG* gene editing strategy through Cri-dots.

Bacterial relapses and recurrent infections are related to the ability of UPEC to form biofilms. Biofilm formation enables bacteria to persist in vagina and bladder, raising the risk of recurrent UTI^61^. Bacterial antibiotic resistance in the biofilm communities imparts to the chronic infections, which leads to the spread of chronic drug resistance bad bugs^62^. Conventional antimicrobials are not effective enough to control biofilm infections and the number of novel therapeutic strategies are still in development^63^. Hence, there is a need for innovative strategies with combination of both anti-biofilm and anti-virulence effects^64^. *papG* has been found to be more prevalent in strong biofilm producer strains^38,39,65^. Also, *papG* is essential for *E.coli* causing recurrent UTI in women^18^. We observed that Cri-dots mediated *papG* targeting significantly diminished the biofilm forming ability of UPEC. Large cellular aggregates of control strain are embedded in exopolymeric matrix, whereas individual cells with small clumps were observed for *papG* targeted strain, which further confirms the reduced adherence and biofilm forming potential of *papG* targeted strain (Fig. 7b). Therefore, *papG* was verified as an important target for developing combination strategy with both anti-adhesive and anti-biofilm effect against UPEC.

In conclusion, this is the first report suggesting use of biocompatible and non-cytotoxic CQDs for CRISPR mediating gene editing in bacteria where Cri-dots can be used as a delivery system for targeting various virulence genes. We showed that the Cri-dots targeting *papG* gene can be successfully delivered into bacteria. A direct role of *papG*, in both adherence and biofilm formation is showed. Therefore, *papG* could be a probable candidate for anti-virulence therapy in drug resistant UPEC. This study might open a new strategy of antimicrobials that can specifically target pathogenic strain without affecting commensal strain. However, further research is required to validate the in vivo efficacy of Cri-dots as a successful therapeutic strategy for UPEC mediated UTIs.

## Materials and methods

### Materials

UPEC strain CFT073 (ATCC # 700928; United States), Commensal strain K12 MG1655 (ATCC # 700926; United States), Luria–bertani broth (Himedia cat # M1245; India), Agar (Himedia cat # GRM666; India), C. elegans Bristol wild type N2 strain , E.coli OP50, THP-1 cells (ATCC # TIB-202; United States), RPMI-1640 (Gibco Cat# 11875119; United States), Hela cells (ATCC # CCL2; United States), DMEM (Cat#L0104, Biowest, Nuaille, France), fetal bovine serum (Gibco Cat# 10270106; United States), T24 cells (ATCC# HTB-4™; United States), McCoy’s 5A modified Media(Gibco Cat# 16600082),3-(4,5-Dimethylthiazol-2-yl)-2,5-Diphenyltetrazolium Bromide-MTT (Invitrogen Cat # M6494; United States), cas9 Protein (Invitrogen Cat # A36497; United States), 1-Ethyl-3-[3-dimethylaminopropyl] carbodiimide hydrochloride (EDC) (Himedia Cat# RM1817, India), N-hydroxysulfosuccinimide (Sulfo-NHS) (Himedia Cat# RM1120, India), HEPES buffer solution (Sigma-Aldrich Cat # 83264; United States), GeneArt™ Precision gRNA Synthesis Kit (Invitrogen Cat # A29377, United States), TRI Reagent (Sigma Cat # T9424; United States), RevertAid First Strand cDNA Synthesis Kit (Thermo Scientific Cat # K1622; United States), SYBR Green Master Mix (Appliedbiosystems Cat# A25742; United States), Crystal violet ( SRL Cat# 28376, India) and SYTO9 green fluorescent nucleic acid stain (Invitrogen Cat # S34854, United States) were used.

### Cell culture

THP-1 cells (Cat # TIB-202, ATCC, Manassas, VA, United States) were cultured in RPMI-1640 medium supplemented with 10% heat inactivated fetal bovine serum, 2 mM L-glutamine, 1 mM sodium pyruvate and 10 mM HEPES (cat#15630080, Life Technologies, Carlsbad, CA, United States). HeLa cells were cultured in Dulbecco’s Modified Eagle Medium (high glucose) supplemented with 10% heat inactivated fetal bovine serum (cat#L0104, Biowest, Nuaille, France) and incubated at 37 °C and 5% CO_2_ in a humidified incubator.

### Bacterial strain and culture conditions

CFT073 known as acute pyelonephritis strain and K12 MG1655, a known commensal strain, were used in this study. It was cultured on Luria-Bertoni (LB) agar or broth medium at 37 °C. OD600 was used to calculate the concentration of bacterial cell culture and MOI (1:5), which was custom used as per requirement of the experiment^66^.

### Cytotoxicity study

#### MTT proliferation assay of THP-1 and Hela cells

CQDs were synthesized by pyrolsis method reported earlier^67^. The cytotoxicity of CQDs was assessed in cell culture using MTT assay. Human monocytic THP-1 cells and HeLa cells were seeded at a density of 2 × 10^4^ cells / well in 100 µl RPMI 1640 and DMEM medium in a 96-well plate at 37 °C in CO_2_ incubator, containing different concentrations of CQDs (0, 12.5, 25, 50, 100 and 200 μg/mL) for 24 h and 48 h. Thereafter, 20 µL MTT (5 mg/mL) in 200 µl of RPMI-1640 medium was added to each well and incubated for 2 h at 37 °C in CO2 incubator. After discarding the 150 µl media, 100 µl of DMSO was added to dissolve the formazan crystals. Next, the optical density was measured at 540 nm using a microplate reader (TECAN Infinity 200 pro). Three independent experiments were performed, and results were presented as the mean ± SEM. One-way analysis of variance (ANOVA) was done to test the significance of difference in the proliferation of THP1 cells at different doses of CQDs using GraphPad’s Prism5 software.

#### MTT assay of Bacteria

CFT073 and K12 was grown overnight in LB medium in a shaking incubator at 200 rpm and 37 °C. The overnight cultures in log phase at OD_600_ between 0.6 and 0.8 (4.8–6.4 × 10^8^ cells/mL) was used for the experiment. Bacterial culture was washed with 1X PBS and diluted 200-fold. Bacteria was incubated overnight with different concentrations of CQDs (0, 12.5, 25, 50, 100 and 200 μg/ml) in a 96-well plate in incubator at 37 °C for 25 h and 48 h. After, standard MTT protocol was followed as described in MTT proliferation assay.

#### Agar Well diffusion method

Antibacterial potency of CQD was analysed against CFT073 and K12 by well diffusion method^68^. Pure cultures of CFT073 and K12 were grown at 37 °C for overnight and plated on sterilized Muller Hinton (MH) plates. Wells of approximately 8 mm diameter were made on MH Agar plate with sterile cork borer. 50 μl of different concentrations of CQDs (0, 12.5, 25, 50, 100 and 200 μg/ml) were loaded to the wells. The plates were thereafter incubated in an incubator at 37 °C for 24 h. The zones of inhibition were examined, and experiments were done in triplicate.

#### Haemolysis analysis

Haemolysis assay was performed as described by Black et al.^69^. Fresh human blood (5 ml) was taken from a healthy volunteer and stabilized with EDTA. The plasma was removed from blood by centrifugation at 3000 rpm for 10 min. Then the RBCs were washed thrice with freshly prepared 150 mM NaCl. Thereafter, the RBCs were diluted by 10% (w/v) with 100 mM sodium phosphate buffer (PBS) (pH 7.4). Diluted RBCs (0.3 ml) were then incubated with different concentrations of CQDs (0, 12.5, 25, 50, 100 and 200 μg/ml) for 3 h at 37 °C. 1% TritonX-100 and 1X PBS served as positive control (PC) and negative control (NC), respectively. After incubation, samples were centrifuged at 3000 rpm for 10 min and 100 ml of supernatant was collected. Absorbance was measured at 541 nm using a UV–vis spectrophotometer. The haemolysis % was calculated according to this formula: Haemolysis % = [(sample absorbance − NC)/ (PC − NC)] × 100, and the average value was obtained from five parallel samples and results were represented using GraphPad’s Prism5 software.

#### Synthesis of Cri-dots (CQD-cas9) conjugates

The standard EDC/NHS conjugation protocol was applied to covalently conjugate carboxyl CQDs to amine group containing cas9 protein. EDC and NHS were mixed in 10 mM (pH 5.5) HEPES buffer at a concentration of 30 and 36 mg/mL, respectively. 10 μl of CQDs (20 μg/ml) were mixed with EDC/NHS mix solution and incubated for 30 min at room temperature to sufficiently activate the carboxyl groups. The carboxyl activated CQD were vortexed in 1 ml of PBST and centrifuged at 6500 g for 30 min. After centrifugation, most of the supernatant was removed. Then, 10 µl of cas9 (8 µg) was added to the mixture and incubated for 4 h at room temperature with mixing. Again, the mixture was thoroughly vortexed in 1 ml of PBST and centrifuged at 6500 g for 30 min. Finally, most of the supernatant was removed, and the solution was dissolved in 50 µl PBS. The conjugate was stored at 4 °C.

### Characterization of CQD and Cri-dots conjugate

#### Transmission electron microscopy

Diluted samples of both CQDs and Cri-dots conjugate were placed on a Carbon-coated 200 square mesh copper grid, air dried and examined using a FEI Tecnai G2-200 kV HRTA transmission electron microscopy (Netherland) operating at 200 kV.

#### Dynamic light scattering

The hydrodynamic diameter of both CQD and Cri-dots conjugate were measured using a Zetasizer Micro V/ZMV 2000 (Malvern, UK). The instrument uses an incident laser beam of 689 nm with a fixed angle of 90°. Set of 3 measurements were made with an acquisition time of 30 s for each sample. The data was analysed using the Malvern Zetasizer Software version 7.01. All measurements were performed at 37 °C.

### Synthesis of single-guide RNA (sgRNA)

#### Designing of gRNA

The gRNA sequences were designed using ‘CHOPCHOP’ webtool^70^. Target site on papG gene was 20 nucleotides in length with adjacent PAM sequence (5′-AGG-3′). The gRNA sequence was designed from the negative strand of targeted gene (Supplementary Fig. S8). On-target gRNA activity was predicted using the CHOPCHOP software analysis, and detailed BLAST searches of *E. coli* genome were conducted to predict any off-target binding of gRNA.

#### In vitro synthesis of gRNA (papG)

In vitro synthesis of gRNA was performed using the GeneArt™ Precision gRNA Synthesis Kit. Oligonucleotide primers for synthesizing gRNAwere purchased from Sigma, with the forward primer containing a T7 promoter sequence. papG gene Specific primers were designed as shown in Supplementary S1 Table. The DNA template for in vitro sgRNA transcription was prepared by assembly PCR using Phusion™ High-Fidelity PCR Master Mix, Tracr fragment, T7 primer mix and 0.3 μM Target F1/R1 oligonucleotide mix according to kit cycling parameters. The DNA template was then in vitro transcribed and purified using gRNA clean up kit. The purified gRNA was quantified using a Nanodrop (ND-1000) and stored at − 80 °C.

#### Cri-dots-gRNA (papG) complex synthesis

CQD-cas9-gRNA(papG) was synthesized using a layer-by-layer method^32^. CQD-cas9 conjugate mixture were mixed with gRNA (2 µg in 10 µl) in 80 µl of HEPES buffer (50 mM, pH 7.5, 300 mM NaCl and 10% glycerol) for 5 min at room temperature. The Cri-dots-papG-nanocomplex complex was stored at − 20° C for the subsequent experiments.

#### Delivery of Cri-dots-papG nanocomplex to CFT073

CFT073 was grown overnight in LB medium in a shaking incubator at 200 rpm and 37 °C. For the experiment, the overnight cultures in log phase at OD600 between 0.6–0.8 (4.8–6.4 × 108 cells/mL) was used for the experiment. Bacterial culture was washed with 1X PBS and 1 ml of culture was incubated overnight with Cri-dots-papG nanocomplex in a 1.5 ml Eppendorf on a rotor spinner (20 rpm). After the treatment, cells were examined for gene targeting efficacy.

### Quantitative real time PCR (qRT-PCR) and mRNA quantification

#### RNA isolation and cDNA synthesis

Total RNA was extracted from bacterial cultures Cri-dots-papG immediately after targeting and control CFT073 using TRI reagent according to manufacturer’s instructions. After quantification using Nanodrop, 1000 ng of total RNA was subjected to cDNA synthesis using cDNA synthesis kit (RevertAid) as per manufacturer’s protocol.

#### qRT-PCR

The gene expression levels of papG were determined by qRT-PCR for control, only CQDs, cas9-papG and Cri-dots-papG. SYBR Green Master Mix was used with conditions as 50 °C for 5 min, 95 °C for 10 min, 40 cycles of 95 °C for 15 s and 60 °C for 1 min in ABI 7300 Real Time PCR machine.16S rRNA and papG gene primers were designed using Primer 3 plus (Supplementary S2 Table). 16S rRNA was used as the reference gene. Relative quantification values were expressed using the fold change method (DDCT) normalized to the control non-targeted samples and results were represented using GraphPad’s Prism5 software.

### Adherence assay

#### Qualitative analysis using Giemsa stain

Adherence assay was performed as described by Giron et al^71^ with some modifications. HeLa cells were seeded at a density of 0.5 × 106 cells / well in a 6- well plate at 37 °C in DMEM supplemented with 10% FBS for overnight. HeLa cells were infected with CFT073 for 3 h at 1:5 MOI (multiplicity of infection, Cells:Bacteria). After 3 h of infection, media was discarded, and cells were washed three times with PBS (1X). Then, cells were fixed with 70% methanol and air dried. Later, they were stained with 10% Giemsa stain for 30 min. The stained plates were then examined by light microscopy at 20X (Motorized Inverted Microscope. Ii2; Nikon). For morphological assessment of cells, HeLa cells were infection with both CFT073 and Cri-dots-papG for 6 h at 1:5 MOI.

#### Quantification analysis using flow cytometry

Adherence assay was performed as described in the previous section. HeLa cells were infected in duplicates with CFT073 and Cri-dots-papG for 3 h at 1:5 MOI. After 3 h infection, media was discarded, and cells were washed three times with PBS to remove unadhered bacterial cells. Cells were then treated with 1 ml of 0.1% tritonX-100 to specifically lyse the mammalian cells. Later, one duplicate was stained with SYTO9 for 10 min in dark and one duplicate remains unstained. For flow cytometry analysis, cells were thoroughly and quickly washed with pulse spin and analysed by FACS Calibur (BD Biosciences) using unfixed cell suspension. The acquisition time for the passage of the sample was set at 60 s, and the event number (number of particles passing through the laser beam) was measured as the number of bacteria in 12 µL of sample. Forward scatter (FSC) and florescence (FL1-Green = 530 / 30 nm) were detected using Linear amplification and flow rate was set at low (12 µL / min). The data were analysed using Flowing software 2.5.1^67,72–74^ and quantitative analysis was done using Prism software (GraphPad) .

#### Haemagglutination assay

Hemagglutinating activity was used for the determination of expression of the papG II and Type 1 fimbrial adhesins. For this, CFT073, Cri-dots—papG and K12 were grown on Luria Bertani plates at 37 °C for 24 h, suspended and serially diluted in PBS (10^10^ bacteria/ml). A suspension of 1% fresh human group O Rh positive erythrocytes from healthy volunteer was mixed with bacterial suspension and added to slide. Haemagglutination was observed both in presence and absence of 1% D-mannose after 2 min. Wells containing only the suspension of erythrocytes were utilized as negative control. Slides were viewed under a microscope (Motorized Inverted Microscope. Ii2; Nikon).

### Biofilm analysis

#### Crystal violet (CV) assay

Biofilm formation was estimated by CV assay as described by Schiebel et al. with some modifications^75^. The procedure was followed as described in the supplementary file for antibiofilm activity determination of CQDs (see supplementary Note S1). Each experiment was performed in triplicate with three independent experiments and analysed using Prism software (GraphPad).

#### Florescence microscopy

For microscopic visualization, biofilms were made as described in the antibiofilm activity determination of CQDs section. After removing planktonic growth from a 96 well plate, 3 µM SYTO 9 green fluorescent nucleic acid stain was added to each well and incubated for 15 min in dark at room temperature. Finally, plate was washed again with PBS (1X) and viewed under a fluorescence microscope (Motorized Inverted Microscope. Ii2; Nikon) using fluorescence setting for FITC (green/SYTO 9). Quantitative analysis of florescence images was carried out using ImageJ software (NIH)^76^ and using GraphPad Prism software.

#### Scanning electron microscopy

Morphological changes of control and Cri-dots-papG bacterial cultures were examined using scanning electron microscopy (EVO18 SEM, Zeiss) with 15 kV voltage. Culture pellets were washed twice with 1X PBS (pH 7.4) and fixed overnight at 4 °C in a mixture of 2% paraformaldehyde and 2.5% glutaraldehyde in 0.1 M phosphate buffer (pH 7.4). After washing, samples were dehydrated with 30, 50, 70, 80, 90, and 100% of acetone for 10 min each. For visualization, samples were spread on an aluminium stubs drop by drop, air-dried and sputter-coated with colloidal gold.

#### *C. elegans* cytotoxicity assay

*C. elegans* cytotoxicity assay was performed as described by Engelsoy et al^77^. CFT073, K12, Cri-dots-papG and *E. coli* OP50 were grown overnight in MSM at 37 °C. 1 × 106 CFU/mL of overnight cultures were grown in MSM in a 96-well plate at 37 °C for 6 h and then cooled down to 21 °C prior to addition of *C. elegans*. Age synchronized L4 worms (Bristol wild type N2 strain) were added to the wells and incubated with bacteria for 1 h at 21 °C. Worms scored as dead or alive and were considered dead when it failed to respond to touch. The data is presented as survival % of *C. elegans* and considered 100% survival in *E. coli* OP50 using GraphPad Prism software.

### Statistical analyses

Statistical analyses were conducted using GraphPad’s Prism5 software. A Student’s *t* test was conducted for two-sample analyses and a one-way analysis of variance (ANOVA) with post-hoc Tukey’s honest significant difference was conducted for multiple sample analyses.

## Supplementary Information

Supplementary Information.

## Data Availability

Data available within the manuscript.
