# Author Correction: Targeting of *Uropathogenic Escherichia coli papG* gene using CRISPR-dot nanocomplex reduced virulence of UPEC

**DOI:** 10.1038/s41598-021-98859-z

**Published:** 2021-09-21

**Authors:** Surbhi Gupta, Parveen Kumar, Bhawna Rathi, Vivek Verma, Rakesh Singh Dhanda, Pooja Devi, Manisha Yadav

**Affiliations:** 1 Dr. B. R. Ambedkar Center for Biomedical Research, University of Delhi, New Delhi, India; 2 Department of Urology, University of Alabama at Birmingham, Hugh Kaul Genetics Building, Birmingham, AL USA; 3 Stem Cell Laboratory, SMiLE Incubator, Scheelevägen 2, Lund, Sweden; 4 CSIR-Central Scientific Instruments Organisation, Sector‑30C, Chandigarh, India; 5 Department of Clinical Sciences, Lund University, Malmö, Sweden

Correction to: *Scientific Reports* 10.1038/s41598-021-97224-4, published online 07 September 2021

The original version of this Article contained a repeated error in the Results section, under the subheadings ‘Cri-dots mediated editing of *papG* reduced the biofilm forming ability of CFT073’ and ‘SEM confirmed a reduced biofilm forming capability in *papG*-targeted CFT073’, where

“Fig. 4”

now reads:

“Fig. 5”

In addition, under the subheading ‘Decreased Virulence of CFT073 after targeting *papG* using Cri-dots’,

“Fig. 5”

now reads:

“Fig. 6”

The original Article has been corrected.

