# Supplementary material for: Targeting of Uropathogenic Escherichia coli papG gene using CRISPR-dot nanocomplex reduced virulence of UPEC
Source: Sci Rep. 2021 Sep 7;11:17801. doi: 10.1038/s41598-021-97224-4 (PMC8423837; doi:10.1038/s41598-021-97224-4)
Supplement: Supplementary file 1 — Supplementary Information. [file 41598_2021_97224_MOESM1_ESM.docx]

**Targeting of *Uropathogenic Escherichia coli* *papG* gene Using CRISPR-dot Nanocomplex reduced Virulence of UPEC**

**Surbhi Gupta^a^, Parveen Kumar^b^, Bhawna Rathi^a^, Vivek Verma^a^, Rakesh Singh Dhanda^c^, Pooja Devi^d^ & Manisha Yadav^a,e*^**

^a^Dr. B. R. Ambedkar Center for Biomedical Research, University of Delhi, New Delhi, India.

^b^Department of Urology, University of Alabama At Birmingham, Hugh Kaul Genetics Building, Birmingham, AL, United States.

^c^Stem Cell Laboratory, SMiLE Incubator, Scheelevägen 2, Lund, Sweden.

^d^[CSIR-Central Scientific Instruments Organisation, Sector-30C, Chandigarh](https://www.microbiologyresearch.org/search?option1=pub_affiliation&value1=%22CSIR-Central+Scientific+Instruments+Organisation%2C+Sector-30C%2C+Chandigarh%22&option912=resultCategory&value912=ResearchPublicationContent), India.

^e^Department of Clinical Sciences, Lund University, Malmö, Sweden.

*Address Correspondence to Manisha Yadav, manisha.dhanda@gmail.com

Short title – CRISPR-dots targeting virulence factor *papG* in UPEC


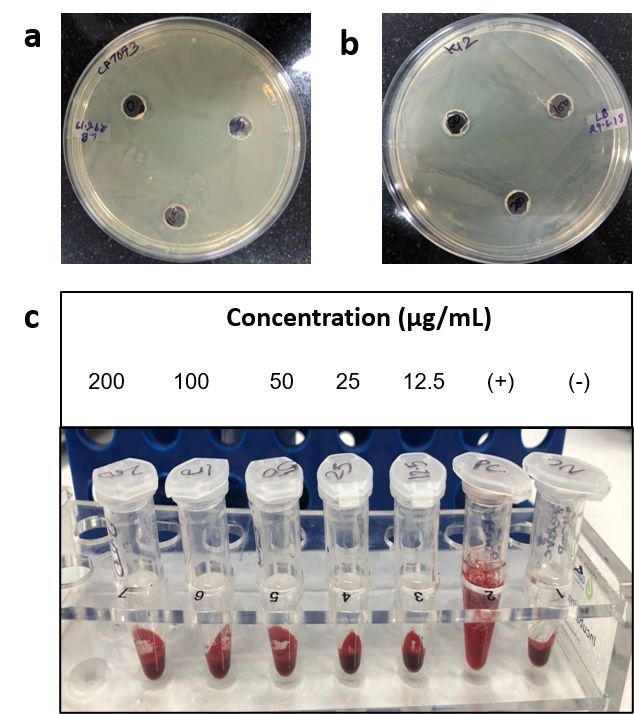


**Supplementary Figure S1. Photographic images**.

Agar well diffusion method for viability testing of CQDs on (a) CFT073 and (b) K12. (c) Tubes containing diluted RBC’s after exposure to different concentration of CQDs.


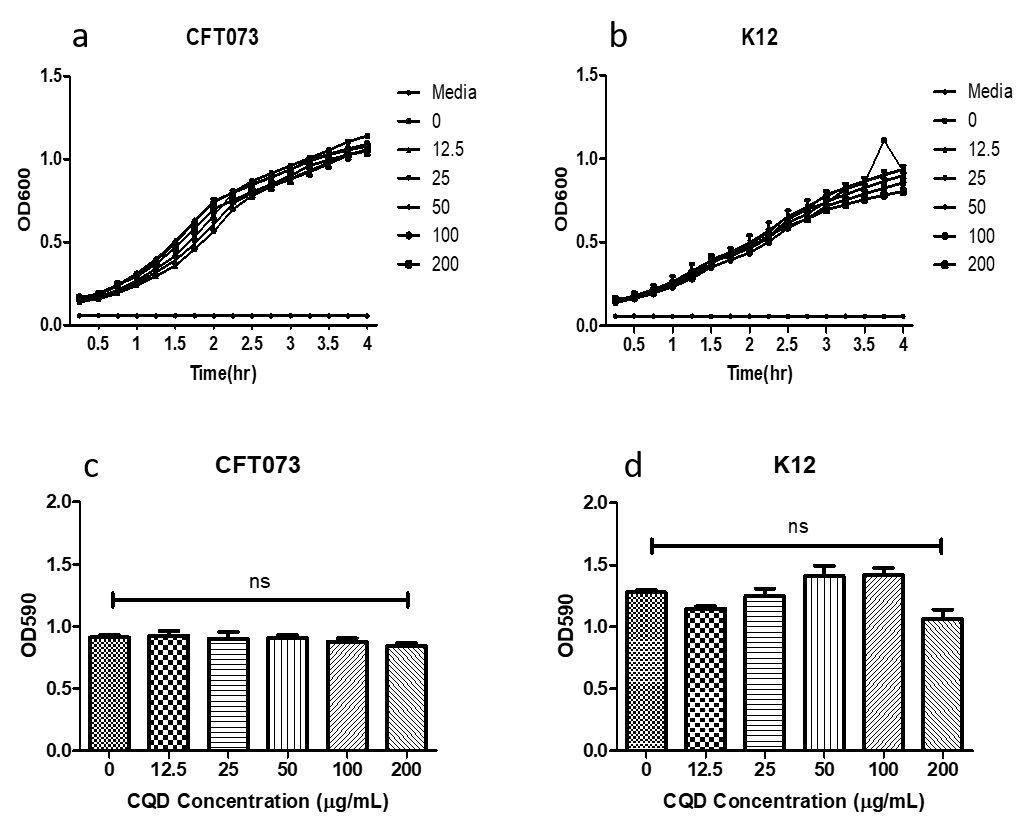


**Supplementary Figure S2. Effects of CQDs labelling on growth and biofilm formation of CFT073 and K12.**

Growth curves for the (A) CFT073 and (B) K12, showing a log of optical density at 600 nm versus time in hr for incubations with different concentration of CQDs (12.5, 25, 50, 100 and 200 μg/ml). CV assay for the (C) CFT073 and (D) K12, showing a log of optical density at 590 nm versus effect of different concentration of CQDs (12.5, 25, 50, 100 and 200 μg/ml) compared to the control with no CQD (0). Data are presented as mean ± SEM of n = 3 independent experiment.


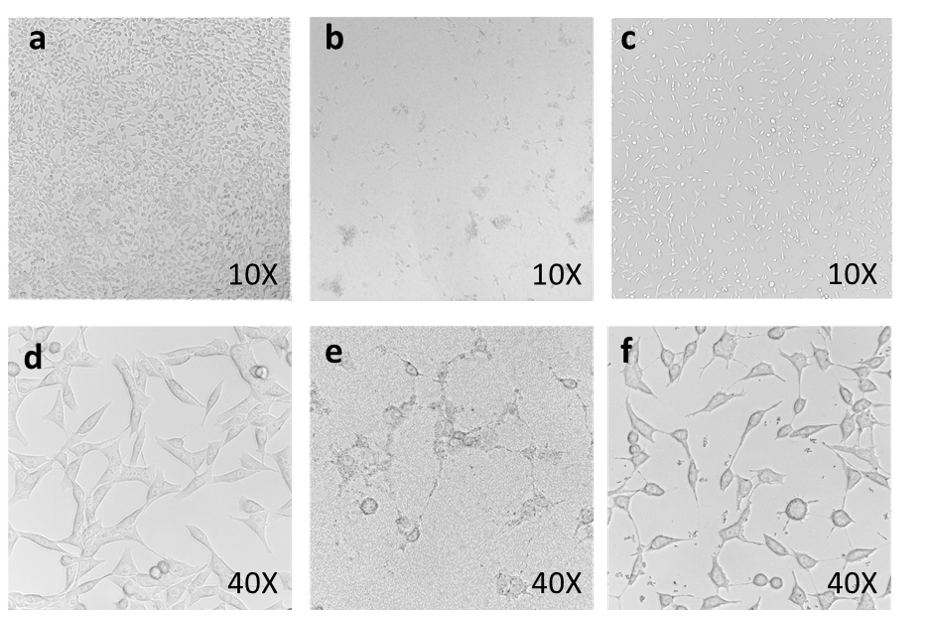


**Supplementary Figure S3. Cell morphology analysis of HeLa cells.**

After infection at MOI 1:5 for 6 hrs. B &C) control CFT073 and C & F)Cri-dots-papG A & D) Healthy HeLa cells.


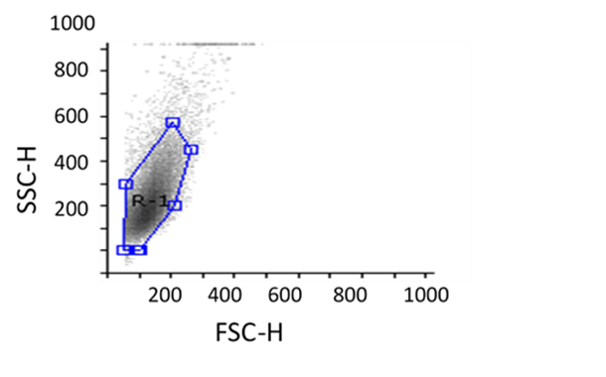


**Supplementary Figure S4. Flow cytometric gating strategy on density plot of unstained CFT073 cells.**

**
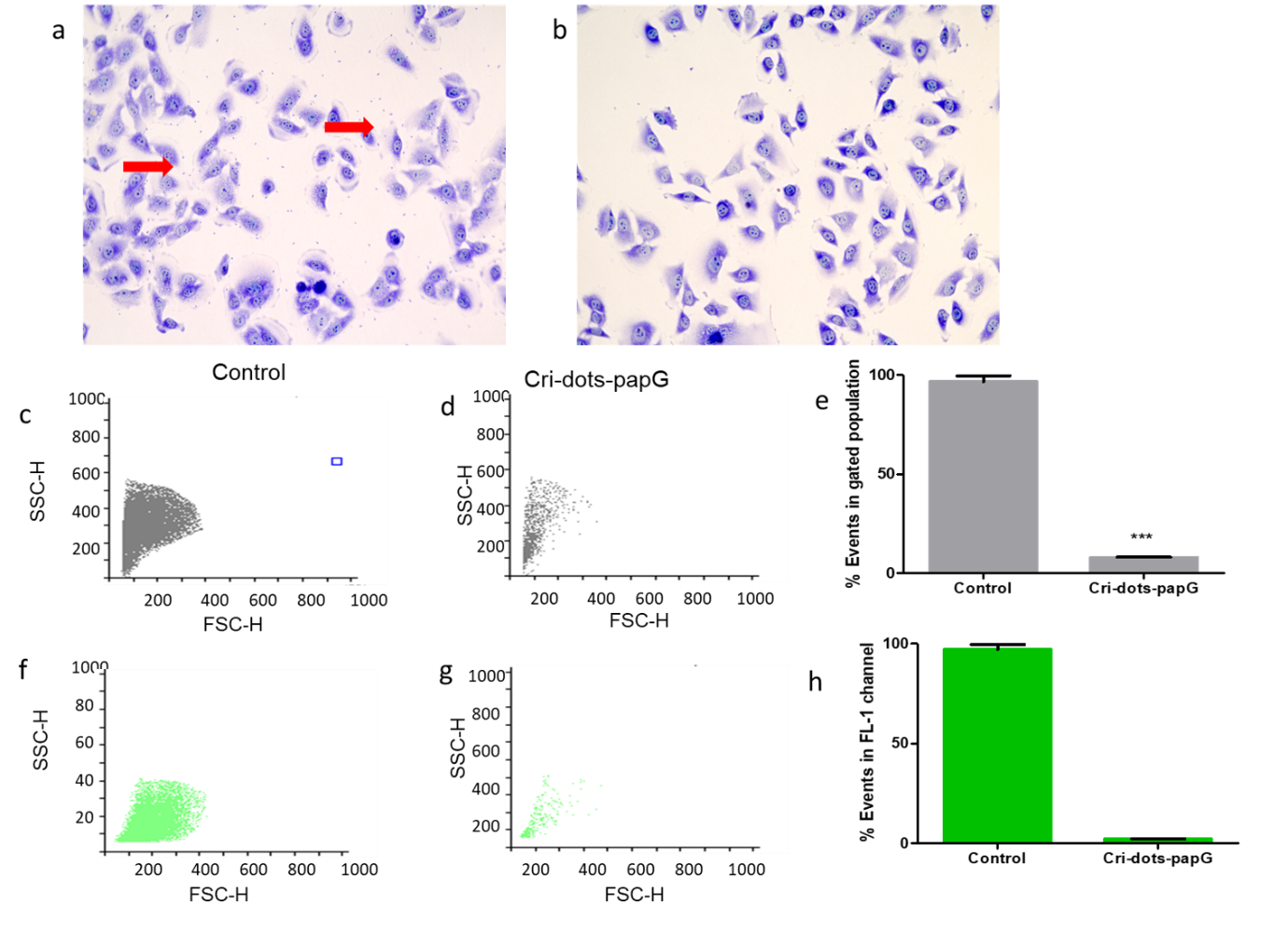
**

**Supplementary Figure S5. Cri-dots-papG targeting CFT073 reduced adherence property to T-24 cells.**

(a-b). T24 cells were infected with (b) Control CFT073 and (c) Cri-dots-papG CFT073 at MOI of 1:5 . At 3 hrs post-infection, the association of bacteria with T24 cells was visualized by Giemsa staining. Arrows indicate bacteria associated with the T24 cells. The plates were examined at 20X. Flow cytometric analysis of adhesion property of Control and Cri-dots-papG CFT073 to t24 cells (c -h). Total numbers of events were acquired in 12 µL of sample volume in 60 sec for each FCM analysis. Panels represents dot plots of gated population of unstained cells (c, d) and SYTO9 cells ( f, g). Panel e and g represents the corresponding % of number of events of bacterial cells normalized to control. The data represents an average of triplicate experiments as mean ± SD. (***P < 0.001, t-test, 2 sided).

**
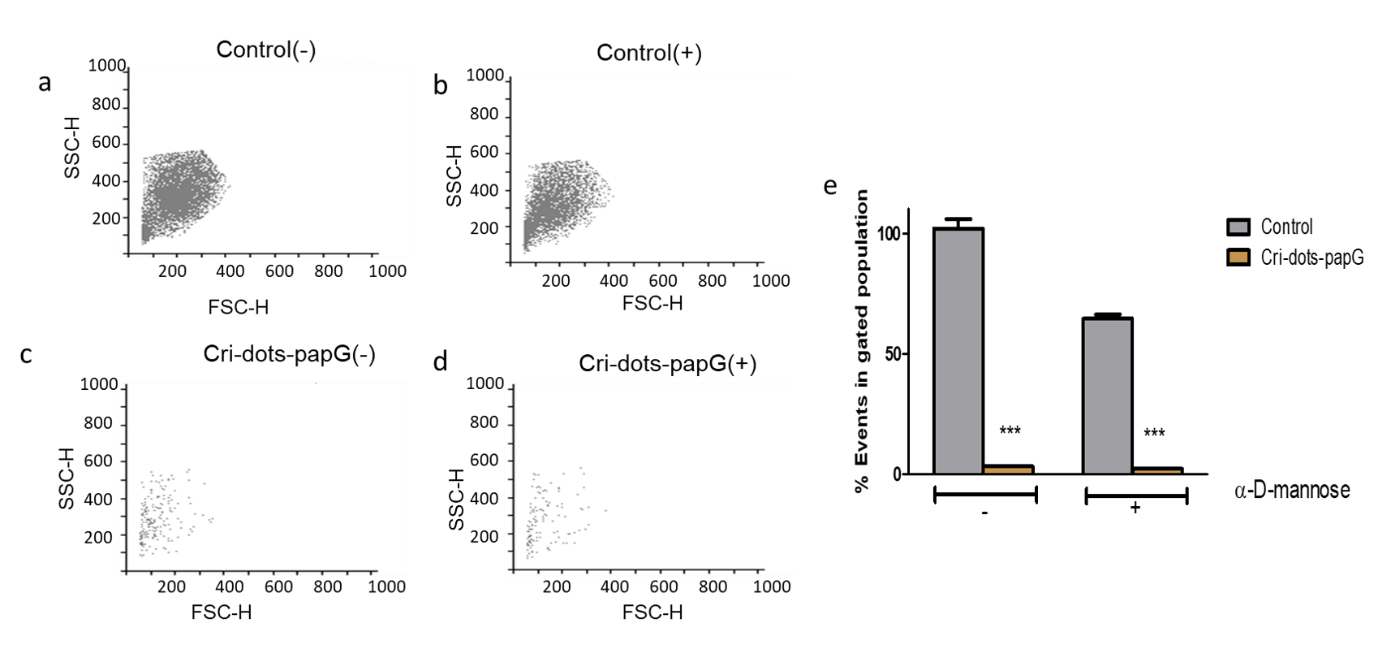
**

**Supplementary Figure S6. Flow cytometric analysis of adhesion property of Control and Cri-dots-papG CFT073 to t24 cells in absence (-) and presence of 1% mannose (+).**

T24 cells were infected with Control CFT073 and Cri-dots-papG CFT073 at MOI of 1:5 in absence (-) and presence of 1% mannose.Total numbers of events were acquired in 12 µL of sample volume in 60 sec for each FCM analysis. Panels represents dot plots of gated population and Panel e represents the corresponding % of number of events of bacterial cells normalized to control. The data represents an average of triplicate experiments as mean ± SD. (***P < 0.001, t-test, 2 sided).

**
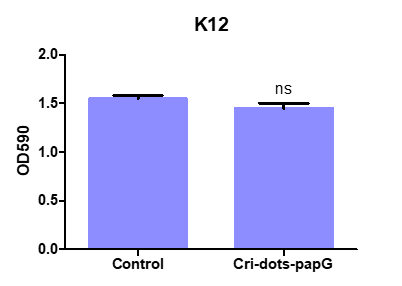
**

**Supplementary Figure S7. Quantification of biofilm formation of K12 was assessed in control and Cri-dots-papG by Crystal Violet assay.**

The data represents an average of triplicate experiments as mean ± SD (2.738 ± 0.07156, control vs, 2.699 ± 0.08130 Cri-dots-papG). (p~ 0.668, t-test, ns).


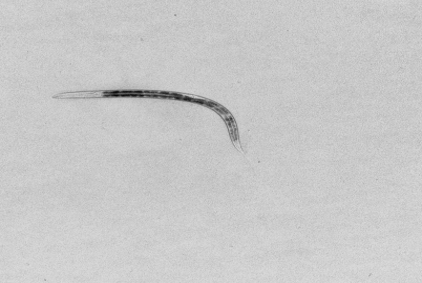


**Supplementary Figure S8. Representative image of *C. elegan*s after infection.**

**
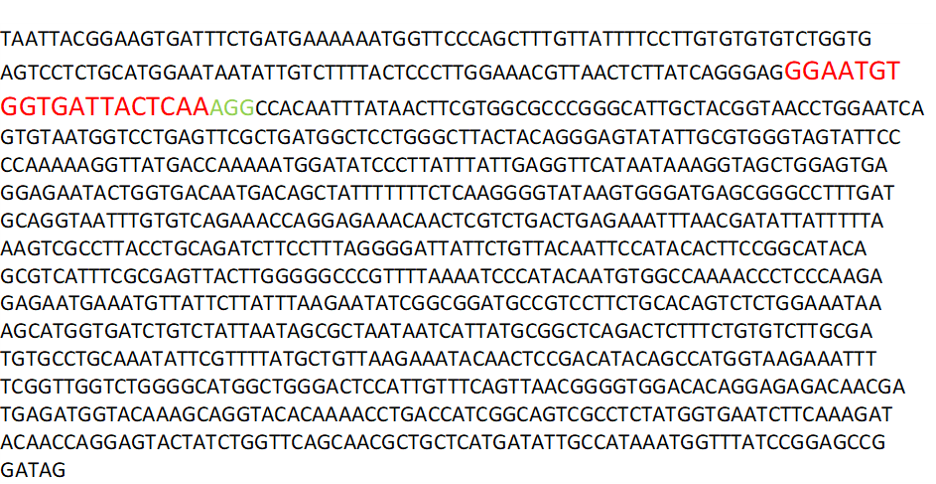
**

**Supplementary Figure S9. Represents the position of gRNA targeting the *papG* gene.**

The gRNA is a 20bp sequence targeting to the Negative strand of *papG* gene that is labelled with red from the Negative strand of *papG*. Green represents the PAM sequence.

| gRNA Forward | TAATACGACTCACTATAGGAATGTGGTGATTACTC |
| --- | --- |
| gRNA Reverse | TTCTAGCTCTAAAACTTGAGTAATCACCACATT |

**Supplementary Table S1. Primers used for gRNA synthesis.**

| papG Forward | GATTCACCATAGAGGCGACTGC |
| --- | --- |
| papG Reverse | GAAATACAACTCCGACATACAGCC |
| 16s rRNA Forward | GCTCGTGTTGTGAAATGTTGGG |
| 16s rRNA Reverse | CTTATGAGGTCCGCTTGCT |

**Supplementary Table S2. Primers used for *papG* gene RT-PCR.**

**Supplementary Note S1. Methods and results of effects of CQDs labelling on growth and biofilm formation of CFT073 and K12.**

**Bacterial growth kinetics at different concentrations of CQDs**

Growth curves were obtained to measure QD toxicity in liquid medium and to determine the effect of CQDs on bacterial growth^1^. *E. coli* CFT073 and K12 culture was inoculated with fresh colonies and incubated for overnight at 37°C in LB media. 1∗10^7^ CFU/mL of overnight cultures were incubated with different concentration of CQDs (12.5, 25, 50, 100 and 200 μg/ml) in a 96-well plate at 37°C for 4 hrs. Curves were obtained by measuring the optical density at 600 nm at each time point from 15 min to 4 hrs using a microplate reader (TECAN Infinity 200 pro) with continuous shaking.

**CQDs didn’t affect the growth of CFT073 and K12**

The growth curve of*CFT and K12* treated with CQDs were determined by using LB supplemented with 12.5, 25, 50, 100 and 200 μg/ml of CQDs. Growth kinetics of both CFT073 and K12 were not affected by the presence of CQDs (Supplementary Fig. S2a and b). The usual sigmoidal growth was observed at all concentration of CQD for both CFT073 and K12.

**Antibiofilm Activity Determination of CQDs**

Anti-Biofilm formation was estimated by CV assay with some modifications^1^]. Overnight cultures of CFT073 and K12 isolates were diluted 1:100 in 200 µl of LB medium and incubated with different concentration of CQDs (12.5, 25, 50, 100 and 200 μg/ml) in a 96 well plate. Bacteria were incubated at 37°C for 24 h without shaking with only media as negative control and bacteria without CQDs as positive control. After incubation, media was removed and washed three times with PBS (1X) solution to remove planktonic growth. The plate was then heat fixed at 60°C for 1 hr. Further adherent biofilms were stained with 200 μl of 0.1% crystal violet solution at room temperature for 10 mins and washed again three times with PBS (1X) solution. The stained biofilms were then solubilised with ethanol 95% v/v for 10 mins and Optical density was measured at 590 nm with a plate reader (TECAN Infinity 200 pro). Each experiment was performed in triplicate with three independent experiments.

**CQDs didn’t affect the biofilm formation of CFT073 and K12**

The effect of the CQDs on the ability of E. coli to form biofilms was measured by crystals violet assay. CFT073 and K12 biofilms were incubated with 12.5, 25, 50, 100 and 200 μg/ml CQDs for 24 h (Supplementary Fig S1c,d). CQDs did not disperse the biomass of both CFT073 and K12 biofilms and can be considered as non-toxic.

1. Kloepfer, J. A., Mielke, R. E. & Nadeau, J. L. Uptake of CdSe and CdSe/ZnS quantum dots into bacteria via purine-dependent mechanisms. *Appl. Environ. Microbiol.* **71**, 2548–2557 (2005).
